# Cochlear implant and congenital cholesteatoma

**DOI:** 10.1186/s40463-016-0119-5

**Published:** 2016-02-01

**Authors:** J. Mierzwinski, AJ Fishman, T. Grochowski, S. Drewa, M. Drela, P. Winiarski, I. Bielecki

**Affiliations:** Department of Otolaryngology, Audiology and Phoniatrics, Children’s Hospital of Bydgoszcz, Chodkiewicza 44, 85-667 Bydgoszcz, Poland; Department of Otolaryngology, Head and Neck Surgery, University Hospital of Bydgoszcz, Ujejskiego 52, 85-168 Bydgoszcz, Poland; Department of Pediatric Otolaryngology, University Children’s Hospital of Katowice, ul Medyków 16, 40-752 Katowice, Poland

**Keywords:** Congenital cholesteatoma, Cochlear implantation, Cochlear implant candidacy, Laser surgery

## Abstract

**Background:**

The occurence of cholesteatoma and cochlear implant is rare. Secondary cholesteatomas may develop as a result of cochlear implant surgery. Primarily acquired cholesteatoma is not typically associated with congenital sensorineural hearing loss or cochlear implant in children. The occurrence of congenital cholesteatoma during cochlear implant surgery has never been reported before, partly because all patients are preoperatively submitted to imaging studies which can theoretically exclude the disease.

**Case presentation:**

We have reported a rare case of congenital cholesteatoma, found during sequential second side cochlear implantation in a 3-year-old child. The child underwent a computed tomography (CT) scan and magnetic resonance imaging (MRI) at 12 months of age, before the first cochlear implant surgery, which excluded middle ear pathology. The mass was removed as an intact pearl, without visible or microscopic violation of the cholesteatoma capsule. All the areas where middle ear structures were touching the cholesteatoma were vaporized with a laser and the cochlear implant was inserted uneventfully. Further follow-up excluded residual disease.

**Conclusion:**

We believe that primary, single stage placement of a cochlear implant (CI) with simultaneous removal of the congenital cholesteatoma can be performed safely. However, to prevent recurrence, the capsule of the cholesteatoma must not be damaged and complete laser ablation of the surface, where suspicious epithelial cells could remain, is recommended. In our opinion, cholesteatoma removal and cochlear implantation should be staged if these conditions are not met, and/or the disease is at a more advanced stage. It is suspected, that the incidence of congenital cholesteatoma in pediatric CI candidates is much higher that in average pediatric population.

## Background

Cholesteatoma is an uncommon condition that has been rarely associated with cochlear implantation. Primary acquired cholesteatoma is not typically associated with congenital sensorineural hearing loss (SNHL) or CI in children. In case of secondary acquired cholesteatomas – they can develop as the result of cochlear implant surgery due to a breach of the posterior wall of the ear canal from drilling the posterior tympanotomy [1]. The identification of congenital cholesteatoma during CI surgery is unlikely because of thorough pre-operative imaging studies, most commonly involving high-resolution computed tomography (HRCT) and MRI of the temporal bone, which can theoretically exclude congenital cholesteatoma. The incidence of congenital cholesteatoma in the overall population is 0.00012 % and 1–3 % of childhood cholesteatomas are congenital [2, 3]. Chung et al. reported that congenital cholesteatoma was identified in 2 out of 794 pediatric CI patients during their pre-operative evaluations for CI (incidence, 0.25 %) [4]. The authors suggest that the incidence was much higher than expected of this rare condition.

Congenital cholesteatoma was initially described by Cawthorne and Griffith [5]. In 1965, Derlacki and Clemis defined congenital cholesteatoma as an embryologic residue of epithelial tissue behind a normal tympanic membrane in the absence of a history of infection or ear surgery [6]. Levenson added that the presence of uncomplicated acute otitis media does not exclude congenital cholesteatoma [7].

Congenital cholesteatoma usually grows slowly as a spherical-shaped keratin-filled cyst in the middle ear with a long asymptomatic period. When early detected, they are located deep to the antero-superior part of the tympanic membrane in two-thirds of the cases. The diagnosis is made at an average age of 4.5 years with a male to female ratio of 1:3. There are two types of congenital cholesteatoma, defined according to their location in the middle ear. The first is an isolated pearl located deep to the anterior part of the eardrum, which is believed to result from arrested epidermal formation at 10 weeks’ gestational age. It is suggested that these formations atrophy at approximately 33 weeks of gestational age, or are evacuated through the Eustachian tube. Failure of this mechanism results in this type of congenital cholesteatoma [8]. The second type is located in the posterior part of the middle ear and causes more rapid ossicular destruction and hearing impairment. The origin of this type is thought to be amniotic fluid cells that migrate in the neonate [9]. The theory of congenital cholesteatoma origin assumes that the pathology is present before birth and the diagnosis is most often made by a combination of otoscopy and HRCT. In a completely aerated tympanic cavity absent of any associated soft tissue, HRCT has a high negative predictive value when excluding cholesteatoma [10]. Microsurgical excision is the accepted treatment and associated laser vaporization of contact points has been shown to limit the rate of recurrence [11]. We present a case of congenital cholesteatoma found this time not during diagnostic procedure before CI, but during sequential second side cochlear implantation in a 3-year-old child in spite of prior imaging studies.

## Case presentation

The patient was a female child diagnosed with bilateral SNHL of genetic origin at the age of 8 months. Genetic testing identified a deletion - 35delG in gene GJB2. The patient failed the newborn hearing screen at birth with subsequent diagnostic auditory brainstem responses demonstrating bilateral, severe to profound SNHL. The child initially received hearing aids but presented significant speech delay despite conventional amplification. Referred for consideration for CI, the patient underwent thorough diagnostic testing by a multidisciplinary team as well as imaging evaluation with HRCT and MRI. HRCT of the temporal bones was performed with a standard protocol using a bone algorithm with a slice thickness of 0.625 mm and collimation of 0.3 mm.

**Fig. 1:**
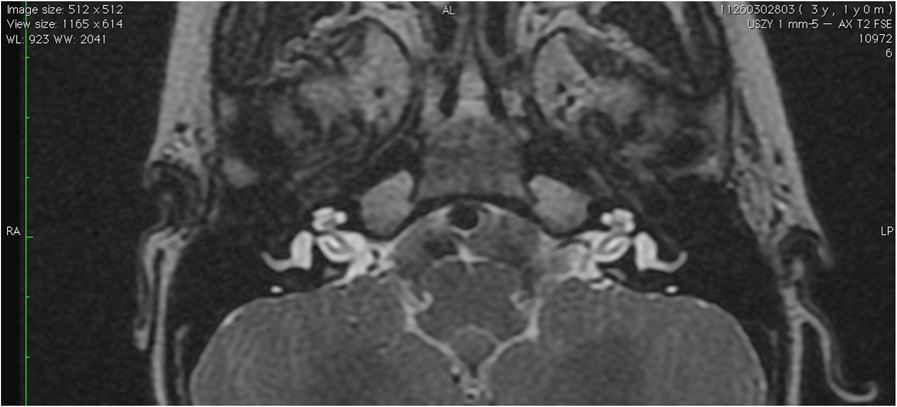
MRI T2-weighted axial image of the ear before the first CI surgery at 1 year of age showing no middle ear pathology

**Fig. 2:**
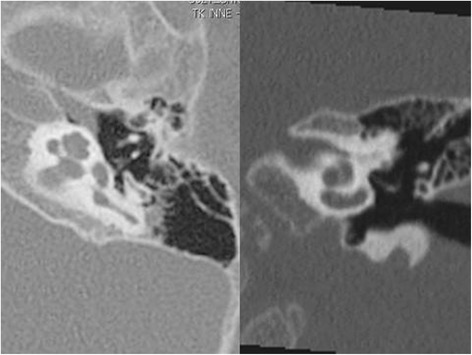
HRCT image (axial-left and coronal-right) of the left temporal bone before the first CI surgery at 1 year of age showing no middle ear pathology

**Fig. 3:**
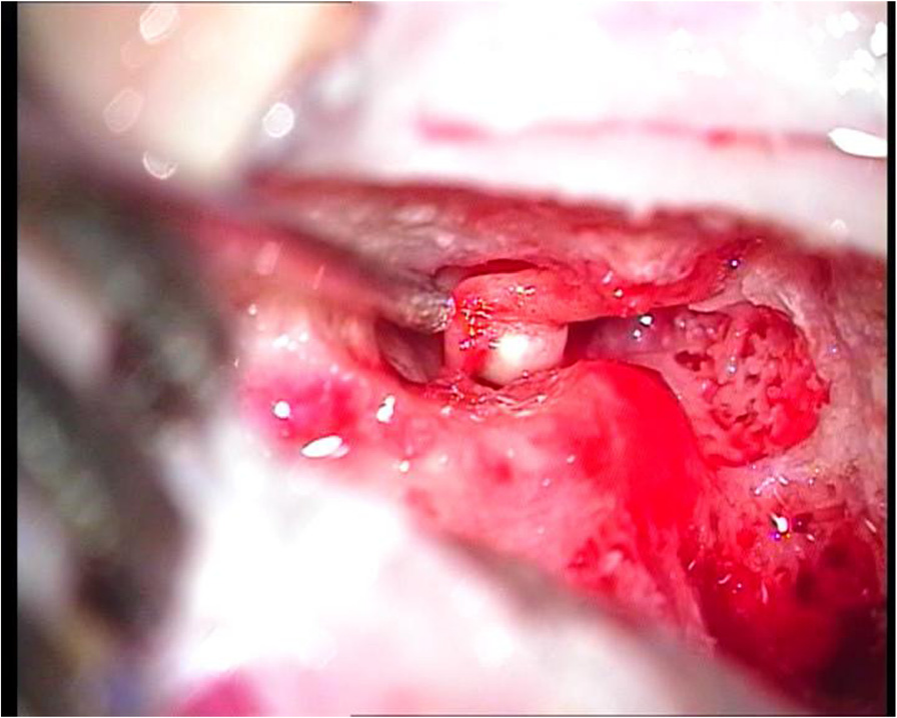
Cholesteatoma, microscopic view

**Fig. 4:**
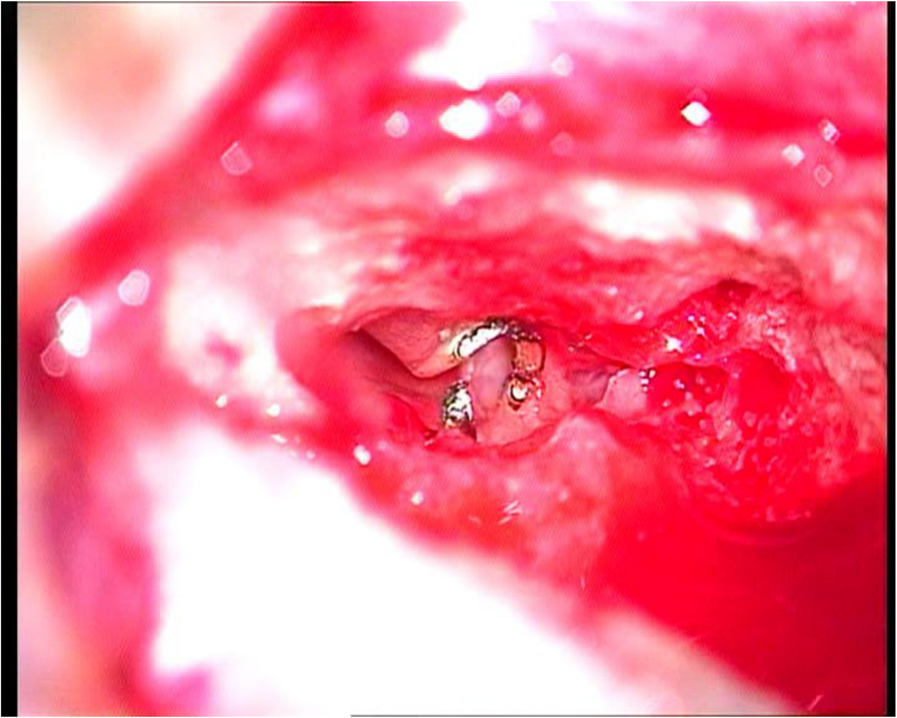
Diode laser treatment of the operated field, microscopic view

Preoperative 1.5 Tesla MRI (Fig. 1) and HRCT (Fig. 2) studies showed implantable inner ear spaces, present cochlear nerves, and no suggestion of additional middle ear pathology. A decision was made to implant the right ear and surgery was performed when the child reached 1 year of age. The surgery on this side was uneventful and without complications or findings of associated middle ear diseases. At the same time, the left ear was equipped with an updated hearing aid. The Integration Scale of Development was used to assess the development of hearing and speech, which is our routine protocol for children between 1 and 4 years of age. The results showed that the child achieved excellent speech and language outcomes comparable to age-appropriate normal hearing subjects. The patient was subsequently evaluated for sequential implantation of the left ear, showing unremarkable otoscopy and a normal tympanic membrane. Normal appearance of the tympanic membrane was also confirmed during otomicroscopy intraoperatively at the time of the second CI surgery. The second implantation was performed two years after the first CI surgery when the child was 3 years old. In our clinic, in cases of sequential implantation, we do not routinely re-image the temporal bones if the first examination showed no pathology. The second CI surgery was performed as per our routine protocol using a posterior tympanotomy approach to the round window and promontory. After opening the facial recess, an approximately 3-mm pearl-appearing cholesteatoma was identified between the facial ridge, incudostapedial joint, and cochleariform process (Fig. 3). To remove the pathology en bloc, the ossicles were disarticulated and the cholesteatoma was removed together with the incus. All tissues contacting the cholesteatoma were vaporized superficially with a diode laser at a setting of 2 W with single short pulses of 0.05 s (Fig. 4) delivered through a 0.6-mm fiber. In surgery for isolated cholesteatoma pearls, we routinely use a laser to minimize the risk of recurrence. The CI was then inserted uneventfully through an extended round window approach.

One year after, a CT of the temporal bone was performed to exclude residual disease and suspicious opacification in the facial recess area was revealed. The residual disease was excluded by the endoscopy of the middle ear through anterior tympanotomy approach. The opacification turned out to be connective tissue used for obliteration of posterior tympanotomy during the CI surgery.

## Discussion

Imaging before cochlear implantation is used to confirm the presence of an implantable inner ear space and intact cochlear nerve, as well as to provide important information about the surgical anatomy of the ear [12]. Both MRI and HRCT can potentially diagnose a pathologic mass such as cholesteatoma in the middle ear space. HRCT has excellent sub-millimeter spatial resolution, which provides accurate delineation of even very small cholesteatomas, as long as there is a well-aerated middle ear cavity. HRCT in this setting offers high sensitivity and excellent negative predictive value [13]. In our case, the conditions for evaluation of the middle ear were excellent. The middle ear was completely free of effusion (Fig. 2), however HRCT has poor specificity because the nature of the soft tissue density cannot be differentiated.

MRI using the conventional sequences (T1-weighted image, T2-weighted image, post-contrast T1-weighted image) provides additional information enabling distinguishment of different pathologic entities, as well as accurate diagnosis of primary and residual/recurrent cholesteatomas. Even higher diagnostic specificity is achieved with diffusion-weighted (DW) echo-planar imaging, delayed post-contrast imaging, DW-non-echo-planar imaging, and DWI-PROPELLER techniques. We used a 1.5 Tesla MRI with conventional sequences in our patient, following our routine protocol, which was focused primarily on assessing the anatomical implant feasibility in young children. However, even with conventional protocols, a pathological mass greater than 2 mm in size and surrounded by air can be identified on the T2-weighted sequence. In conventional MRI, the diagnosis of sub-millimeter anatomical structures is also possible provided that there is good signal contrast between the structure and its surroundings, as in our case. For temporal bone diagnosis in clinical practice, we use a 1.5 Tesla MRI. With the development of new models, MRI at 3 Tesla or higher is becoming more common and widely available. The main advantage is shorter acquisition time but because of artifacts specific to this anatomic region, diffusion-weighted sequence acquisition is paradoxically longer, which increases the risk of motion artifacts, especially in children, making interpretation more difficult, even for experienced radiologists [14].

Except for paper of Chung et al., to date, there has been no literature published on the incidence of congenital cholesteatoma found prior to implantation [4]. The authors found this incidence (0,25 %) much higher than expected of this rare condition in general population (0.00012 %). It is surprisingly common, given the absence of any cases of primarily acquired cholesteatoma in the reported group of patients, which is considerably more common in the pediatric population. Our case, as well as both reported by Chung et al. of congenital cholesteatoma patients, most likely had an inherited form of hearing loss and, as they suggested, genetic contribution to the presence of congenital cholesteatoma cannot be excluded [4]. A correlation between the formation of congenital cholesteatoma and abnormal cochleovestibular anatomy and SNHL have also been reported by Propst et al. and Jackler et al. [15, 16].

It has been suggested that if congenital cholesteatoma is found during diagnostic procedures for CI, the cholesteatoma should be removed and implantation delayed to the second stage [4]. In our patient, we made the decision to remove the cholesteatoma and insert an implant in a one-stage procedure because the disease was removed as an intact pearl, without visible or microscopic violation of the cholesteatoma capsule, and the areas of contact between the cholesteatoma and middle ear structures were vaporized with a laser. The risk of recurrence was very unlikely. Such a protocol has been used in our clinic for several years in numerous ear operations, in cases of limited congenital cholesteatoma and small cholesteatoma pearls found during second-look procedures. We consider the procedure safe and do not hesitate to proceed with ossicular reconstruction in such cases. What is more, revision surgery is also not planned in such situations.

Although the laser is not universally utilized in the treatment of cholesteatoma, Hamilton concludes that the appropriate use of the laser during cholesteatoma surgery facilitates significantly the complete removal of the disease and presents no extra risk to the vital structures within the temporal bone [11]. Also James et al. stated that current technological advances such as laser and middle ear endoscopy contribute to better outcomes in the treatment of cholesteatoma [17]. As it has been mentioned we routinely use laser during cholesteatoma surgery in our clinic.

It has long been recognized that a second-look procedure in cases of congenital cholesteatoma is required less often than in acquired pediatric cholesteatoma [18]. James et al. suggested that when the cholesteatoma cyst is removed intact, complete eradication can be almost guaranteed, and recommended a second-look procedure when cholesteatoma extends into hidden regions such as the mastoid, or with concerns about the completeness of matrix removal [17]. We also follow this strategy. In our case, the cholesteatoma did not extend into hidden spaces; it was a closed capsule and we had no concerns about incomplete removal. It should be emphasized that the decision to proceed with CI requires careful consideration on a case-by-case basis. Particularly with a larger congenital cholesteatoma, a damaged capsule, or widespread pathology, delaying CI is the most reasonable and safest option [4].

According to Derlacki and Clemis’ criteria the patient’s cholesteatoma met the definition of a congenital cholesteatoma - it was totally asymptomatic and behind an intact tympanic membrane [6]. Cholesteatoma was not seen at otoscopy before the first and second surgery. Before the first surgery it was most probably so small that it was not within the limits of visibility even for CT and MRI. The cholesteatoma was not seen at otoscopy at the second surgery either because it was located medially to the long crus of the incus and the handle of the malleus. Middle ear pathology located in the posterior mesotympanum is practically invisible until it touches the tympanic membrane and can be easily overlooked.

The growth rate of this particular cholesteatoma had to be relatively dynamic.

The original size of the cholesteatoma is important. According to the first theory of origination of congenital cholesteatoma, the diameter of the epidermoid formations frequently found in human fetuses that proceed to congenital cholesteatoma if not absorbed or evacuated through the Eustachian tube, is already known. Huang et al. reviewed 49 fetuses, ranging from 12 weeks to full term. In 16 they found epidermoid formation, which was always located at the anterosuperior edge of the eardrum [19]. The width and height of the epidermoid formations was 60.82 ± 5.68 microns and 45.87 ± 6.82 microns, respectively. In our case, the cholesteatoma was located in the posterior mesotympanum, and met the second theory of origination, which says that amniotic fluid cells migrate through the Eustachian tube to the middle ear and then form the cholesteatoma. In this case, the cholesteatoma might have arisen from a single cell, or group of cells, also microns in diameter.

Assuming a linear growth for congenital cholesteatoma, James et al. calculated that closed cholesteatoma cysts enlarge by approximately 1 mm in diameter per year, based on CT measurements ([20] James). We can assume in our case that the growth was linear because it was a closed round capsule; therefore, in the first year of life, theoretically, the cholesteatoma would have been approximately 1 mm in diameter, enlarging to approximately 3 mm in diameter at 3 years of age. A 1-mm pearl would be visible by imaging studies and the negative predictive value of CT in such a case is excellent [6].

The explanation for the lack of identification of the cholesteatoma on the initial HRCT is that the pathology was too small to be visualized. The most likely it was a very small sub-millimeter “sleeper” congenital cholesteatoma with no growth between birth and the first year, but with subsequent rapid growth between 1 and 3 years of life.

Bilateral cochlear implantation in young children is increasingly common in clinical practice. Among the benefits of bilateral cochlear implantation is the restoration of some of the advantages of binaural hearing such as localization, improved listening in noise, directional hearing, binaural summation, and squelch. Typically, young hearing-impaired children are being provided with two implants either at the same time (simultaneous implantation) or at different times in early childhood as in our case (sequential implantation) [21]. Our local public funding policy enables children to receive initially unilateral cochlear implant due to economic limitations. In our clinic, excluding the post meningitis deafness, when bilateral implants are put simultaneously, the CI candidates are implanted unilaterally as quickly as possible, starting from the age of 12 months. Only when we are able to provide all candidates with one implant and meet the economic limitations, do we consider giving another implant to prior pediatric CI users.

Examining the findings in this patient, the obvious question arises whether to perform another imaging study which could preclude the existing, newly formed cholesteatoma before the subsequent CI surgery, and how much time should elapse between the first and second implantation, to perform such a study most effectively?

We believe that the rarity of our particular case does not justify the additional cost and burden of repeating the standard HRCT and/or MRI in so young children. Also, our case presented with limited pathology that was controlled by a single-stage procedure. We emphasize the importance of continued follow-up and advise considering staging in either known disease or more extensive disease as a reasonable and safe option.

## Conclusions

To our knowledge, this is the first report of an incidental finding of congenital cholesteatoma during CI surgery despite presurgical imaging studies. Primary excision en bloc with the removal of associated ossicular elements and laser surface ablation is the recommended treatment for limited congenital cholesteatoma. Primary placement of an implant during cholesteatoma removal is warranted as long as there is insignificant risk of recurrence, provided that no damage of the capsule has occurred and complete and safe surface contact of epithelial laser ablation was observed. There should be a low threshold for staging the implant if any of these conditions are not met, and/or the disease is found to be more extensive. Follow-up should include regular microscopic ear examination and HRCT.

There is suspicion that the incidence of congenital cholesteatoma in pediatric CI candidates is much higher than in normal pediatric population (4).

### Consent

The written consent was obtained from the patient’s parents to publish the details of their child’s case.

## Abbreviations

CT: Computed tomography
MRI: Magnetic resonance imaging
CI: Cochlear implant
SNHL: Sensorineural hearing loss
HRCT: High resolution computed tomography
DW: Diffusion-weighted
